# Molecular Characterization, Evolution and Expression Analysis of Ammonium Transporter from Four Closely Related *Bactrocera* Species (Tephritidae)

**DOI:** 10.3390/life14091114

**Published:** 2024-09-04

**Authors:** Jie Zhang, Qi Wang, Chenhao Liu, Jiaying Liu, Qian Qian, Chuanjian Ru, Leyuan Liu, Shanchun Yan, Wei Liu, Guirong Wang

**Affiliations:** 1 School of Forestry, Key Laboratory of Sustainable Forest Ecosystem Management-Ministry of Education, Northeast Forestry University, Harbin 150040, China; jiezhang666@foxmail.com (J.Z.); qwang218@163.com (Q.W.); liuchenhao98@163.com (C.L.); qianqian120224@163.com (Q.Q.); rcj20000913@163.com (C.R.); 2 College of Tropical Agriculture and Forestry, Hainan University, Haikou 570228, China; jyliu211@163.com; 3 College of Plant Health & Medicine, Qingdao Agricultural University, Qingdao 266071, China; le8825@126.com; 4 Shenzhen Branch, Guangdong Laboratory of Lingnan Modern Agriculture, Key Laboratory of Synthetic Biology, Ministry of Agriculture and Rural Affairs, Agricultural Genomics Institute at Shenzhen, Chinese Academy of Agricultural Sciences, Shenzhen 518120, China; wangguirong@caas.cn

**Keywords:** Ammonium Transporter, olfactory receptor, Tephritidae

## Abstract

Numerous insects are attracted to low levels of ammonia, utilizing it as a cue to locate food sources. The Ammonium Transporter (Amt), a highly conserved, atypical olfactory receptor, has been shown to mediate the detection of ammonia in insects. While the attraction of Tephritidae to ammonia is well established, knowledge about the *Amt* in this family is limited. The species *Bactrocera dorsalis* (Hendel 1912), *Bactrocera cucurbitae* (Coquillett 1899), *Bactrocera correcta* Bezzi 1916 and *Bactrocera tau* (Walker 1849), which are common agricultural pests within Tephritidae, exhibit numerous ecological similarities, offering a solid foundation for studying *Amt* characteristics in this family. In this study, we elucidated the sequences, evolutionary relationships, and expression patterns of *Amt* in these four species. The results indicated that these *Amts* share the same open reading frame, containing 1770 bp that encode a protein of 589 amino acid residues. These Amt proteins exhibit the typical structural characteristics of Amts, including an 11-transmembrane domain with an extracellular N-terminus and an intracellular C-terminus. They also have the ability to form trimers in the membrane. Additionally, they contain three conserved amino acid residues essential for ammonia transport: A189, H195, and H352. Phylogenetic and expression pattern analyses showed that they are highly conserved in Diptera and are significantly expressed in antennae. This study is the first report characterizing the Amt gene in four Tephritidae species. These findings provide a foundation for further exploration into the roles of these genes in their particular biological contexts.

## 1. Introduction

The Tephritid fruit fly (Diptera: Tephritidae) is a group of insects that severely damages fruits and vegetables [1]. Comprising over 4600 species, this family is widely distributed across tropical, subtropical, and temperate regions [2]. The primary source of damage from Tephritid fruit flies is attributed to the females. Females lay their eggs within fruits or vegetables, causing mechanical damage and promoting microbial infections. The emerging larvae then feed internally, leading to the premature drop and decay of the fruits and vegetables. Consequently, the affected agricultural products become inedible, and stringent quarantine measures further impede the export of fruits and vegetables, resulting in significant economic losses [3]. Among these fruit flies, the genus *Bactrocera* is notably destructive, primarily found in the tropical and subtropical regions of Asia [4]. Common species within this genus include *Bactrocera dorsalis* (Hendel 1912), *Bactrocera cucurbitae* (Coquillett 1899), *Bactrocera correctan* Bezzi 1916, and *Bactrocera tau* (Walker 1849).

Protein is a crucial nutrient throughout the growth and development of Tephritid fruit flies. To support the development of their reproductive organs, adult females require a substantial amount of this nutrient [5,6,7,8,9,10]. Without sufficient protein, females are unable to lay eggs [11]. In their natural habitats, fruit flies obtain protein from host plants, bacteria, and secretions of bacteria [12]. However, these minor proteins and their hydrolyzed amino acids lack strong volatility, hindering insects from pinpointing these protein sources across vast distances. Consequently, fruit flies have developed sophisticated localization strategies to secure an adequate supply of protein-rich food; they depend on the volatile scents emitted from the decomposition of proteins as indicators to find protein-abundant food sources [12,13]. Ammonia and its derivatives serve as typical indicative scents. Leveraging this trait, early agricultural workers conceived ammonia-derivative-based lures that have proven effective in managing Tephritidae populations [14,15,16]. Therefore, ammonia compounds hold considerable ecological significance for fruit flies.

In the natural environment, insects predominantly rely on their highly sensitive olfactory systems to detect volatiles released by hosts or food sources [17]. Identifying olfactory molecular targets for key compounds is crucial in the development of olfaction-based behavioral modifiers, as it forms the fundamental basis for such advancements [18]. Despite numerous accounts of the attractant effects of amine compounds on flies, a regrettable gap in research exists regarding their olfactory recognition mechanisms. Fortunately, studies have elucidated the olfactory recognition pathways for ammonia in mosquitoes and *Drosophila*. Research has revealed that Ammonium Transporter (Amt), an atypical olfactory receptor, significantly influences the transport of ammonia in antennae [19,20,21]. Amt is a ubiquitous transmembrane protein found across various organisms, including bacteria, fungi, plants, and animals [22,23,24,25]. It features 11 transmembrane domains and forms a functional trimeric structure on the membrane. These proteins display a pronounced affinity for ammonium ions (NH_4_^+^) and are instrumental in mediating their transmembrane transport. In both *Drosophila melanogaster* Meigen 1830 and *Anopheles gambiae* Giles 1902, the *Amt* gene exhibits high expression in the antennae and directly facilitates the response of specific neurons within the antennal sensors to ammonia. This represents the first known instance of a transport protein functioning as an olfactory receptor in animals [19,20,21]. Although Amt is also expressed in the taste organ labellum of *Drosophila*, it does not appear to mediate the gustatory perception of amines [26]. Introducing the *A. gambiae Amt* into *Drosophila* mutants deficient in *Amt* effectively reinstates the electrophysiological response of neurons to ammonia, and orthologs of *Amt* have been found in many insect species [19,20,21]. Notably, for larger molecular amines produced by protein degradation, such as putrescine and cadaverine, there are no reports indicating the involvement of *Amt* in their perception. Based on this evidence, we hypothesize that the function of *Amt* may be conserved among insect species. Nevertheless, there are currently no reports on the function of the *Amt* gene in Tephritid fruit flies.

In this study, utilizing the peripheral transcriptomes of *B. dorsalis*, *B. cucurbitae*, *B. correcta*, and *B. tau*, we analyzed the amino acid domain structure of their *Amt*, coupled with an evolutionary comparison with closely related species. We also delineated the expression profiles of these genes across various olfactory organs and body regions. Our aim is to provide a foundational basis for future research into the functional role of the Amt gene in olfactory recognition.

## 2. Materials and Methods

### 2.1. Insect Rearing and Sample Collection

The wild-type laboratory strains of *B. dorsalis*, *B. cucurbitae*, *B. correcta*, and *B. tau* used in this study were provided by the Agricultural Genomics Institute at Shenzhen, Chinese Academy of Agricultural Sciences. These flies were reared on an artificial diet at 26 ± 1 ℃ and 60 ± 10% relative humidity under a 14 L/10 D photoperiod. The larval diet comprised banana, yeast, sucrose, corn flour, and cellulose paper. Upon maturity, larvae were transferred to wet sand for pupation. After pupation completion and sand drying, pupae were collected and placed in insect cages (18 cm × 24 cm × 14 cm) for emergence. Three days before emergence, males and females were separated and housed in smaller cages (18 cm × 12.5 cm × 14 cm). Adults were fed an artificial diet of yeast and sugar (1:1) with an additional water source.

At 12 days post-emergence, peripheral sensory tissues (antennae, mouthparts, maxillary palps, legs, and external genitalia) from both sexes were collected. Each replicate included 100 individuals, with a total of three replicates. Samples were immediately cryopreserved in liquid nitrogen and stored at −80 °C until further use.

### 2.2. RNA Isolation and cDNA Synthesis

Total RNA was extracted from various tissues using TRIzol reagent (Invitrogen, Carlsbad, CA, USA), and its integrity and concentration were assessed by agarose gel electrophoresis and a NanoDrop 1000 spectrophotometer (Thermo Scientific, Wilmington, DE, USA). Approximately 1 µg of total RNA was used as a template for cDNA synthesis with the HiScript^®^ III 1st Strand cDNA Synthesis (+gDNA wiper) Kit (Vazyme, Nanjing, China). The resulting cDNA was diluted and stored at −20 °C for subsequent PCR use.

### 2.3. Homology Search and Molecular Cloning

Utilizing the previously acquired peripheral transcriptome data of *B. dorsalis* (China National GeneBank Database: PRJCA020830), *B. cucurbitae* (unpublished), *B. correcta* (unpublished), and *B. tau* (unpublished) from our laboratory, we independently assembled transcriptomes for each *Bactrocera* with Trinity-v2.8.5 [27] and identified candidate coding regions using TransDecoder v5.5.0. We downloaded the *D. melanogaster* complete genome protein sequence from NCBI and performed homology alignment with Orthofinder v2.5.4 [28] against the transcript protein sequences of four *Bactrocera* species. From these alignments, we selected candidate *Amts* homologous to *D. melanogaster Amt* (*DmelAmt*) for each species. We performed a multiple sequence alignment using DNAMAN and predicted the secondary structures of these candidate *Amts* with DeepTMHMM (https://services.healthtech.dtu.dk/services/DeepTMHMM-1.0/, accessed on 16 June 2024). We compared the nucleic acid sequences of the candidate *Amts* from the four *Bactrocera* species to confirm the completeness of all candidate *Amt* structures and to eliminate intron retention.

We designed specific primers based on the predicted gene sequences for amplifying the ORFs of four Amts, as detailed in Appendix A. Following PCR, we purified the products using the EasyPure Quick Gel Extraction Kit (TransGen Biotech, Beijing, China) and cloned them into a blunt vector. Sequencing was conducted in both the 5′- and 3′-directions by Sangon Biotech, Shanghai, China.

### 2.4. Bioinformatic Analyses

We used the ProtParam tool from ExPASy (https://www.expasy.org/resources/protparam, accessed from 15 June 2024, to 23 June 2024) to calculate the theoretical isoelectric points, molecular weights, instability indices, and aliphatic indices of the proteins, and we used the ProtScale tool from ExPASy (https://www.expasy.org/resources/protparam, accessed from 15 June 2024, to 23 June 2024) to calculate the average hydropathy indices of the proteins. The tertiary structures were predicted using the AlphaFold Server (https://alphafoldserver.com/, accessed from 15 June 2024, to 23 June 2024), which is based on the advanced AlphaFold 3 model [29]. The accuracy of these predictions was assessed via Ramachandran plot analysis in UCLA-DOE LAB-SAVES v6.0 (https://saves.mbi.ucla.edu/, accessed from 15 June 2024, to 23 June 2024). Protein structure images were generated using PyMOL v2.5 [30].

### 2.5. The Phylogenetic Analysis of the Amt Family

In order to explore the phylogenetic relationships between four *Amts* and documented Amts from various Diptera species, we acquired the Amt amino acid sequences of 16 Diptera species. These species include *Bactrocera tryoni* (Froggatt 1897), *Bactrocera neohumeralis* Hardy 1950, *Bactrocera latifrons* (Hendel 1915), *Bactrocera oleae* (Rossi 1790), *Anastrepha ludens* (Loew 1873), *Ceratitis capitata* (Wiedemann 1824), *Anastrepha obliqua* (Macquart 1835), *Rhagoletis pomonella* (Walsh 1867), *Teleopsis dalmanni* (Wiedemann 1830), *Musca vetustissima* Walker 1849, *Musca domestica* Linnaeus 1758, *Lucilia cuprina* (Wiedemann 1830), *D. melanogaster*, *Aedes aegypti* (Linnaeus 1762), *A. gambiae*, and *Anopheles coluzzii* Coetzee & Wilkerson 2013, all of which were retrieved from the Uniprot database (https://www.uniprot.org/, accessed on 15 April 2024; the accession numbers of the sequences used are listed in Appendix B). Following this, multiple sequence alignment was performed using MAFFT v7.515 [31]. The optimal nucleotide substitution model was determined with ModelFinder in IQ-TREE v2.2.0.3 [32]. A phylogenetic tree was then constructed using the maximum likelihood method.

### 2.6. Analysis of Expression Patterns of Amt in Different Peripheral Tissues

We realigned clean reads from each sample to the *Amt* using HISAT v0.1.6-beta (Hierarchical Indexing for Spliced Alignment of Transcripts) and assessed *Amt* expression in peripheral tissues via FPKM quantification [33,34,35,36]. Differential expression analysis was conducted with R package DESeq2 [37], identifying significant gene expression changes with a *p*-value ≤ 0.05 and an absolute log2Ratio ≥ 1 [38]. PCR conditions and primer sequences are detailed in Appendix A. The PCR reaction mixture totaled 10 µL, consisting of 5 µL of 2 × Taq Pro Universal SYBR qPCR Master Mix (Vazyme, Nanjing, China), 0.5 µL of each 10 µM primer, 1 µL of cDNA template, and 3 µL of RNase-free water. The amplification process was executed on a CFX96 real-time PCR detection system (Bio-Rad, Hercules, CA, USA). Specificity was confirmed by dissociation curve analysis, and gene expression was quantified by the 2^−ΔΔCT^ method [39].

### 2.7. Statistical Analyses

All replicated data are presented as mean ± standard error. The normality of the raw data was initially assessed using the Shapiro–Wilk test. Non-normally distributed data were transformed using log (*x* + 1). Differences in the data were analyzed using an unpaired *t*-test, with significance considered at *p* < 0.05. All statistical analyses were performed using GraphPad Prism (Version 8.0.1).

## 3. Results

### 3.1. Identification and Sequence Characteristics of Amts

**Figure 1:**
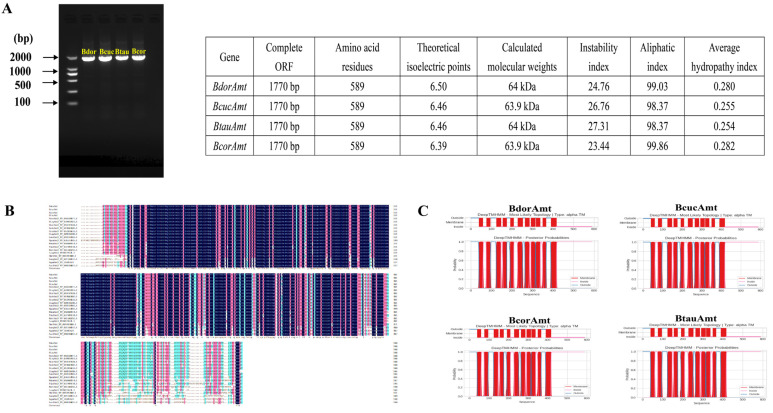
The identification and sequence characteristics of Amts from *B. dorsalis* (BdorAmt), *B. cucurbitae* (*BcucAmt*), *B. correcta* (*BcorAmt*), and *B. tau* (*BtauAmt*). (**A**) Electrophoresis of four *Amt* PCR products and prediction of the physicochemical properties of the encoded proteins. (**B**) Multiple sequence alignment of amino acid sequences of Amts from *B. tryoni*, *B. neohumeralis*, *B. latifrons*, *B. oleae*, *A. ludens*, *C. capitata*, *A. obliqua*, *R. pomonella*, *T. dalmanni*, *M. vetustissima*, *M. domestica*, *L. cuprina*, *D. melanogaster*, *A. aegypti*, *A. gambiae*, and *A. coluzzii*; the accession numbers of the sequences used are listed in Appendix B. (**C**) Transmembrane topology profile prediction of four *Amts*.

Using the transcriptome datasets from four related fruit fly species, we successfully identified the genes *BdorAmt*, *BcucAmt*, *BcorAmt*, and *BtauAmt* by aligning with *DmelAmt* sequence (Table 1). Using their antennae cDNA as templates, we designed specific primers for PCR amplification of these genes (Appendix A). The electrophoresis showed that the amplified target fragment was consistent with the length of the target genes (Figure 1A). These genes are the same length, containing a 1770 bp ORF encoding an Amt protein consisting of 589 amino acid residues (Figure 1A; Appendix C).

The theoretical isoelectric points of BdorAmt, BcucAmt, BtauAmt, and BcorAmt range from 6.39 to 6.50, and their molecular weights range from 63.9 to 64 kDa. Their instability indices are 24.76, 26.76, 27.31, and 23.44; aliphatic indices are 99.03, 98.37, 98.37, and 99.86; and average hydropathy indices are 0.280, 0.255, 0.254, and 0.282, respectively (Figure 1A; Appendix D). To ascertain the completeness of the ORF lengths obtained, we performed multiple sequence alignments and secondary structure predictions. The results of the multiple sequence alignment revealed extensive conserved regions within these proteins (Figure 1B), while secondary structure predictions indicated that all four Amts encompass 11 transmembrane domains, with an apoplastic N-terminus and a cytosolic C-terminus (Figure 1C). These findings confirmed the integrity of the identified Amt ORFs.

### 3.2. Phylogenetic Analysis of Amts

**Figure 2:**
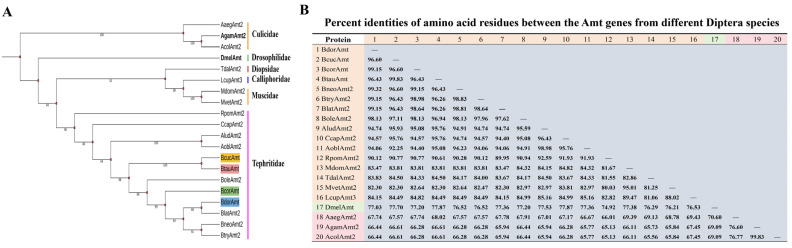
Phylogenetic analysis of Diptera Amts and comparison of amino acid residue similarity; the accession numbers of the sequences used are listed in Appendix B. (**A**) Phylogenetic relationships of Diptera Amt sequences. (**B**) Percent identities of amino acid residues between the Amts from different Diptera species.

We analyzed the conservation of *Amt* genes and constructed a phylogenetic tree based on amino acid sequences from Diptera available in public databases. This analysis revealed significant homology among Amt proteins across various Diptera species. The four identified Amt proteins show the highest homology with those from the Tephritidae family, followed in descending order by proteins from several species within the Muscidae, Calliphoridae, Diopsidae, Drosophilidae, and Culicidae families (Figure 2A). Specifically, BdorAmt and BltaAmt clustered together, while BcucAmt and BtauAmt formed another branch (Figure 2A). Detailed amino acid sequence analysis indicated that the Tephritidae Amts shared >90% similarity; the four Amt proteins exhibited sequence similarity exceeding 82% with Amts from *M. domestica*, *T. dalmanni*, *M. vetustissima*, and *L. cuprina*; and each demonstrated a sequence similarity of up to 77% with DmelAmt and up to 66% with AgamAmt2 (Figure 2B). It is noteworthy that DmelAmt and AgamAmt2 have been implicated in the olfactory recognition of ammonia [19,20,21]. Additionally, other Amts from fruit flies exhibited over 66% homology with both DmelAmt and AgamAmt2 (Figure 2B). Such a high level of sequence similarity suggests a conserved functional role for this gene family.

### 3.3. Structural Prediction of Amt and Analysis of Conserved Functional Sites

**Figure 3:**
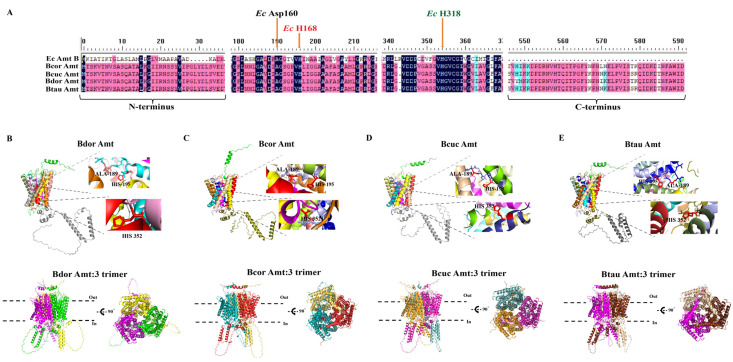
In silico analyses of Amt protein sequence from *B. dorsalis* (BdorAmt), *B. cucurbitae* (BcucAmt), *B. correcta* (BcorAmt), and *B. tau* (BtauAmt). (**A**) Protein alignment of Amts from *E. coli* and *B. dorsalis*, *B. cucurbitae*, *B. correcta*, and *B. tau*. (**B**–**E**) Protein structure prediction for BdorAmt, BcorAmt, BcucAmt, and BtauAmt.

Amt’s role in the transport of NH_4_^+^ across various organisms, including bacteria, fungi, plants, and insects, has been well established [22,23,24,25]. To predict the functional roles of BdorAmt, BcucAmt, BtauAmt, and BcorAmt, we analyzed their structural features and conserved functional sites. In *Escherichia coli* (Migula, 1895), extensive research has elucidated the functional residues of EcAmt [22,23]. Building on this knowledge, we conducted a comprehensive multiple sequence alignment focused on EcAmt to identify highly conserved residues critical for the functionality of the four Amts. Our analysis revealed three conserved positions corresponding to previously documented functional residues in EcAmt: Ec Asp160, Ec H168, and Ec H318. Specifically, Ec Asp160 aligns with A189 in the four Amts, Ec H168 with H195, and Ec H318 with H352 (Figure 3A). Notably, Ec Asp160, Ec H168, and Ec H318 from the *E. coli* homolog, EcAmtB, have been confirmed to play a crucial role in ammonium ion transport [22,23].

To delve deeper into the structural features of the four Amt proteins, we used AlphaFold 3 to predict their tertiary structures (Figure 3B–E). The Ramachandran favored values were all above 90% (Appendix D), indicating the validity of the predicted three-dimensional structures. The characteristics of their 2D structures align with those of the 3D structures. Another important functional characteristic of the Amt proteins is their ability to form trimers on the cell membrane [22,23,24,25], and we predicted a similar trimeric structure (Figure 3B–E, Appendix D).

### 3.4. Expression Profiles of Amts in Different Peripheral Tissues

**Figure 4:**
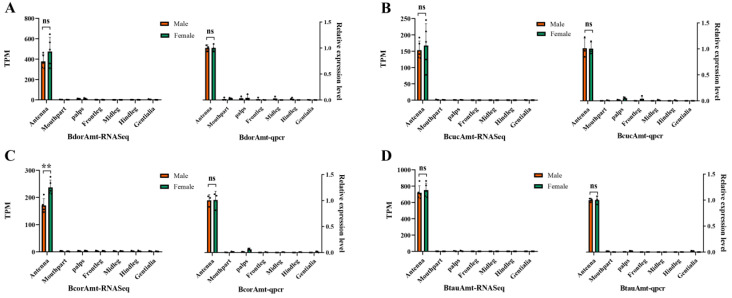
The expression levels of *Amt* in different body parts of *B. dorsalis* (*BdorAmt*, **A**), *B. cucurbitae* (*BcucAmt*, **B**), *B. correcta* (*BcorAmt*, **C**), and *B. tau* (*BtauAmt*, **D**). Each image separately illustrates the results of RNA-Seq quantification and the Quantitative Real-Time PCR (qRT-PCR) verification. Results are presented as the mean ± standard error. *p* values were determined by a two-tailed unpaired *t*-test. (** p < 0.01; ns indicates no significant difference).

We conducted a transcriptome analysis to examine the expression of four *Amt* genes in various peripheral tissues and olfactory organs. We then confirmed these findings with qPCR. The results confirmed the presence of *Amt* transcripts in adult tissues and revealed a highly consistent expression pattern of the *Amt* genes across peripheral tissues in the four fly species. Specifically, high levels of transcription were observed in the antennae, whereas almost no expression was detected in the mouthparts, maxillary palps, legs, and external genitalia (Figure 4A–D). Furthermore, there were no significant differences in the expression of *Amt* genes between males and females in these flies (Figure 4A–D).

## 4. Discussion

Ammonia serves as a mediator for insects to locate protein-rich food sources [12,13]. The function of Amt as an ammonia receptor was first explored in *Drosophila* [19,21]. Subsequently, *AgamAmt2* was reported to have similar functionality in *A. gambiae* [20]. The function of Amt in the olfactory detection of ammonia is hypothesized to be conserved across insects. However, in the economically significant pest genus *Bactrocera*, research on *Amts* remains absent. Therefore, this study reports the sequence information and expression patterns of *BdorAmt*, *BcucAmt*, *BtauAmt*, and *BcorAmt* based on the peripheral transcriptomes from four closely related species, *B. dorsalis*, *B. cucurbitae*, *B. correcta*, and *B. tau*, and predicts the structural characteristics of the amino acids. These findings will allow future study of the physiological functions of these genes.

The structure and functional characteristics of *Amt* genes have been extensively studied in microorganisms and plants [22,23,24,25]. In *E. coli*, the crystal structure of EcAmtB has been reported, showing that it is a protein with 11 transmembrane regions that forms trimers in the membrane. Each monomer contains a hydrophilic channel located between the relatively polar cytoplasmic and periplasmic vestibules, directly involved in the transport of NH_4_^+^ [22,23,24]. Similar reports have also been found in studies on Amt in fungi and plants [22,25]. The structural and functional characteristics of this protein are similar to those of an ammonia transporter protein, RhCG (SLC43A3), in humans, which is a member of the 386 human SLC superfamily members. It also forms a trimeric functional structure and participates in the transport of ammonia in the human kidney. However, it possesses an additional transmembrane domain compared to EcAmtB [40,41]. In this study, based on transcriptome data from four species, we screened and cloned their respective *Amt* genes: *BdorAmt*, *BcucAmt*, *BtauAmt*, and *BcorAmt*. These genes have identical ORF lengths, each comprising 1770 bp and encoding a protein of 589 amino acid residues. Structural predictions revealed that all four Amts possess a typical 11-transmembrane domain structure with an N-terminus outside the membrane and a C-terminus inside and are capable of forming trimers in the plasma membrane. They also contain three conserved amino acid residues, A189, H195, and H352, which are known to be key residues in ammonia transport in *E. coli* [22,23]. These findings suggest a high accuracy in the sequences of the four *Amts* identified, and the predicted structural features suggest potential for transporting NH_4_^+^. The complete sequence information will provide a foundation for future in vitro functional studies.

Through phylogenetic analysis, we found that the four Amt proteins are highly homologous within Diptera, containing numerous conserved segments. Among the Tephritidae species used in this study, sequence similarity between these Amts exceeds 80%. Notably, BdorAmt, BcucAmt, BtauAmt, and BcorAmt show sequence similarity of up to 77% with DmelAmt and 66% with AgamAmt2. Additionally, peripheral olfactory expression profiling showed that all four Amts are predominantly expressed in the antennae. Among these homologous genes, DmelAmt and AgamAmt2 have been demonstrated to be significantly expressed in the antennae and participate in ammonia recognition as atypical olfactory receptors [19,20,21]. It was hypothesized that Amt may directly facilitate the rapid and efficient transport of ammonia/ammonium ions into cells, leading to changes in membrane potential, which may trigger action potentials through an unknown signaling mechanism. In the antennae, highly expressed olfactory proteins often play crucial roles in olfaction [42,43], and highly conserved genes in insects often have similar functions [44,45,46]. The four Amts identified in this study are highly conserved within Diptera and significantly expressed in the antennae, suggesting they may have similar functions to those reported for mosquitoes and *Drosophila*, i.e., participating in ammonia recognition in the antennae. Additionally, previous studies have indicated low *Amt* expression in the labellum of *Drosophila*, yet we found no significant *Amt* levels in the mouthpart transcriptome [26]. This discrepancy likely arises from sampling the entire mouthparts, diluting the labellum signal. Future studies should specifically sample the labellum to obtain accurate measurements. Future research should explore in vitro functional studies using the *Drosophila* empty neuron system [47,48], and in vivo functional validation through knockout experiments.

## 5. Conclusions

In summary, this study has investigated the sequence information, structural characteristics, phylogenetic relationships, and expression patterns of *Amt* genes from *B. dorsalis*, *B. cucurbitae*, *B. correcta*, and *B. tau*. The findings indicate that all four Amt proteins possess the typical Amt transmembrane domain structural features, including three conserved amino acid sites previously reported to be involved in ammonia transport. These residues are highly conserved across Diptera and significantly expressed in the antennae of the four Tephritid fruit flies. We postulate that these Amt proteins might play a role in the antennal recognition of ammonia. Future research is required to validate the in vivo and in vitro functions of these genes.

## Figures and Tables

**Table 1:** Candidate Amt genes of *B. dorsalis*, *B. cucurbitae*, *B. correcta*, and *B. tau*.

Gene Name	Blastx Best Hit (Reference/Name/Species)	E-Value	Score
*BdorAmt*	XP_049304235.1 putative ammonium transporter 2 [*Bactrocera dorsalis*]	0	1211
*BcorAmt*	XP_049304235.1 putative ammonium transporter 2 [*Bactrocera dorsalis*]	0	1204
*BcucAmt*	XP_054081809.1 putative ammonium transporter 2 [*Zeugodacus cucurbitae*]	0	1209
*BtauAmt*	XP_054081809.1 putative ammonium transporter 2 [*Zeugodacus cucurbitae*]	0	1097

**Figure A1:**
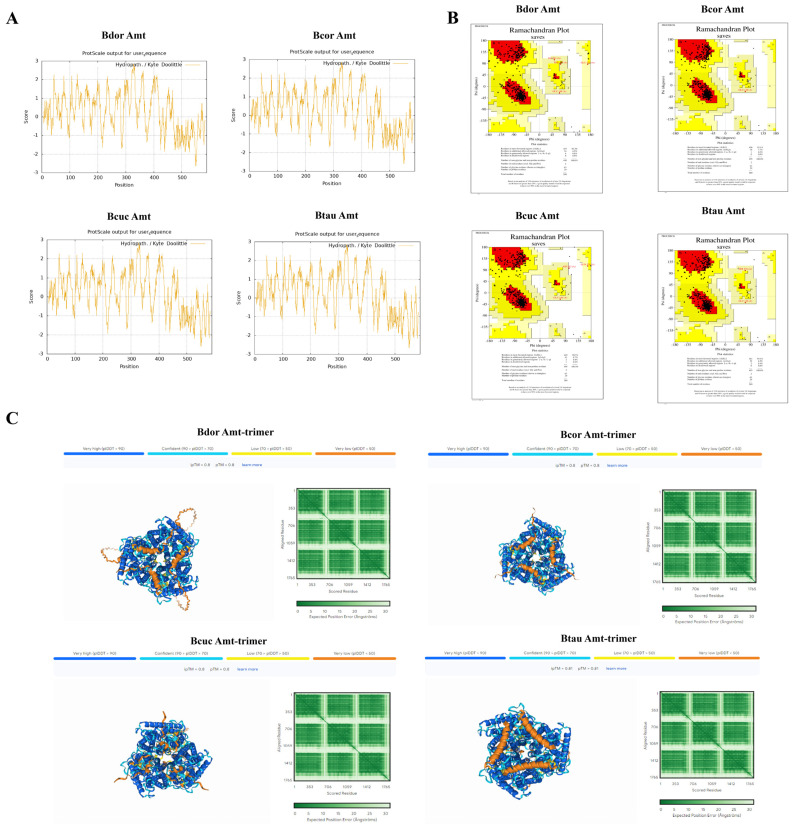
The hydrophilicity/hydrophobicity (**A**) and confidence scores for the predicted structures of *B. dorsalis* Amt (BdorAmt), *B. cucurbitae* Amt (BcucAmt), *B. correcta* Amt (BcorAmt), and *B. tau* Amt (BtauAmt) (**B**,**C**). In (**B**), this Ramachandran plot highlights the various regions of polypeptide chain conformations, the regions are marked as follows: A, Alpha helix region (right-handed α-helix); B, Beta sheet region (β-sheet); L, Allowed regions for other conformations; a, Right-handed alpha helix; b, Antiparallel beta sheet; l, Left-handed alpha helix; p, Parallel beta sheet; ~a, Allowed region for right-handed alpha helix; ~b, Allowed region for antiparallel beta sheet; ~l, Allowed region for left-handed alpha helix; ~p, Allowed region for parallel beta sheet.

## Data Availability

Data are contained within the article. The original contributions presented in the study are included in the article; further inquiries can be directed to the corresponding authors.

## Appendix A

**Table A1:** Summary of primers, PCR conditions.

	PCR Primers	PCR Conditions
*BdorAmt*	BdorAmt-CDS-F: ATGACTTCAAAAGTGACAAATGTCT BdorAmt-CDS-R: TCAATCGATCCATGCAAAATTGATAT	98 °C for 3 min; then 35 cycles of 98 °C for 10 s, annealing at 60 °C for 15 s, extension at 72 °C for 2 min; followed by a final extension of 72 °C for 10 min; stored at 10 °C
*BcorAmt*	BcorAmt-CDS-F: ATGACTTCAAAAGTGACAAATGTCTC BcorAmt-CDS-R: TCAATCGATCCATGCAAAATTGATAT	98 °C for 3 min; then 35 cycles of 98 °C for 10 s, annealing at 60 °C for 15 s, extension at 72 °C for 2 min; followed by a final extension of 72 °C for 10 min; stored at 10 °C
*BcucAmt*	BcucAmt-CDS-F: ATGACTTCAAAAGTGACAAATGTCTCAG BcucAmt-CDS-R: TCAATCGATCCATGCGAAATTGGTAT	98 °C for 3 min; then 35 cycles of 98 °C for 10 s, annealing at 60 °C for 15 s, extension at 72 °C for 2 min; followed by a final extension of 72 °C for 10 min; stored at 10 °C
*BtauAmt*	BtauAmt-CDS-F: ATGACTTCAAAAGTGACAAATGTCT BtauAmt-CDS-R: TCAATCGATCCATGCGAAATTGGTAT	98 °C for 3 min; then 35 cycles of 98 °C for 10 s, annealing at 60 °C for 15 s, extension at 72 °C for 2 min; followed by a final extension of 72 °C for 10 min; stored at 10 °C
*BdorAmt*(q-PCR)	BdorAmt-q-F: GAACACCGGATTCGTCACT BdorAmt-q-R: CGCGTAGCTGTTCAATGCTC	95 °C for 3 min; then 39 cycles of 95 °C for 10 s, annealing at 55 °C for 30 s, +Plate Read; followed Melt Curve 65 °C to 95 °C, increment 0.5 °C, for 5 s; Plate Read
*BcorAmt*(q-PCR)	BcorAmt-q-F: CTTTGTGCTCTGGTGGGGAT BcorAmt-q-R: CATCATGACGCCAAAGCGAG	
*BcucAmt*(q-PCR)	BcucAmt-q-F: CGCCATGATGGACGTATGGA BcucAmt-q-R: CCCCTAACCGGTCGAACAAA	
*BtauAmt*(q-PCR)	BtauAmt-q-F: GCTCGGTTCACTGGTCTCAA BtauAmt-q-R: CCCCTAACCGGTCGAACAAA	
*BdorActin-2*(q-PCR)	BdorActin-2-F: GTGTGATGGTTGGTATGGGA BdorActin-2-R: GGCTGGGGAGTTGAAGGTTT	
*Bcor_GAPDH2*(q-PCR)	Bcor-F: CTTCAACTGGTGCCGCTAAG Bcor-R: ACCCTTCATTGGACCCTCTG	
*Bcuc_GAPDH2*(q-PCR)	Bcuc-F: TGCTACTACTGCCACCCAAA Bcuc-R: ACGGAAAGCCATACCAGTCA	
*Btau_GAPDH2*(q-PCR)	Btau-F: TGCTACTACTGCCACCCAAA Btau-R: ACGGAAAGCCATACCAGTCA	

## Appendix B

**Table A2:** The accession numbers of Amt amino acid sequences used in this study.

Gene Name	Accession Number
*Bactrocera neohumeralis Amt2 (BneoAmt2)*	XP_050328977.1
*Bactrocera tryoni Amt2 (BtryAmt2)*	XP_039952923.1
*Bactrocera latifrons Amt2 (BlatAmt2)*	XP_018797628.1
*Bactrocera oleae Amt2 (BoleAmt2)*	XP_014087802.1
*Anastrepha ludens Amt2 (AludAmt2)*	XP_053956833.1
*Ceratitis capitata Amt2 (CcapAmt2)*	XP_004518340.1
*Anastrepha obliqua Amt2 (AoblAmt2)*	XP_054726403.1
*Rhagoletis pomonella Amt2 (RpomAmt2)*	XP_036333465.1
*Musca domestica Amt2 (MdomAmt2)*	XP_058984514.1
*Teleopsis dalmanni Amt2 (TdalAmt2)*	XP_037949734.1
*Musca vetustissima Amt2 (MvetAmt2)*	XP_061386255.1
*Lucilia cuprina Amt3 (LcupAmt3)*	KAI8129331.1
*Drosophila melanogaster Amt (DmelAmt)*	NP_001097800.1
*Aedes aegypti Amt2 (AaegAmt2)*	XP_021700161.1
*Anopheles gambiae Amt2 (AgamAmt2)*	XP_318439.4
*Anopheles coluzzii Amt2 (AcolAmt2)*	XP_040218851.2

## Appendix C

**BdorAmt**

>amt trans

ATGACTTCAAAAGTGACAAATGTCTCGGCATCCCAAGCCACTGCGTTAAAAGGCATTATCCGCAACAGTAGTTATGTCATACCAGGATTGTACGACCTTAGCGTGGAGGATACCAATTGGGTGTTGACGTCGTCGTTCATCATTTTCACAATGCAAACTGGGTTTGGCATGCTTGAGTCGGGTTGTGTATCGATTAAAAATGAGGTCAACATCATGATGAAGAACGTCATTGATATTGTACTTGGCGGCTTTACCTACTGGCTCTTCGGTTATGGCATGAGTTACGGCCGTGGACCACTTTCGAATCCATTCATAGCAGTTGGAGACTTTCTACTGGATCCGCCTGTGGGTGATCCCTTAATGGGGCAAATTTTTGCTGCATTTTTATTTCAGTTGTCCTTTGCTACGACAGCAACAACGATTGTTAGTGGCGCCATGGCAGAAAGATGCAATTTCAAAGCGTACTGTATATTTTCGCTATTAAATACTGCTGTCTACTGTATTCCCGCCGGTTGGGTGTGGGGTGAACATGGCTTCCTCAATAATCTTGGAGCTGTTGATATAGCGGGAAGTGGACCCGTACATTTAATCGGAGGTGCAGCTGCATTTGCATCTGCTGCCATGTTGGGTCCACGTTTGGGTCGCTACGCAGACGGCTATGATCCTCTGCCACTTGGTAATCCTGTGAACGCATGCATGGGTCTATTTGTGCTCTGGTGGGGATGGCTAGCTTTTAATTCCGGCAGTACATATGGTGTAAGTGGTGCCAAATGGCAATATGCTGCACGTGCTGCTGTCATGACTATGATGGGTTCATTTGGTGGTGGTATCATAAGTTGCGCGTACTCGCTTTGGCGTCATGATGGCCGTATGGATATTATCGATCTTATTAACGGTGTACTCGGGTCATTGGTCTCAATTACGGCTGGGTGTTTTCTATATCGTGCTTGGGAGGCACTAGTCATTGGGATGTTGGGTGCCTTACTTTGTGTTGTCGCAATGCCGTTGTTCGACCGGTTAGGAGTCGACGACCCAGTAGGTGCAAGCTCTGTTCACGGAGTTTGTGGCATTTGGGGTGTTATCGCGGTTGGACTTTTCGCTGACAATCCTGTACCGTTAGAGACAACAAAGGGACGTTCAGGACTATTTAAAGGTGGCGGTTGGTACTTGGTTGGCATACAGACTTTGTCGGCATTATGCCTTGCCTGCTGGGGCATATGTTCAACGTTTCTACTACTTTATATTATCAACAAATTTATACCTTTACGCATGGATCCAAATGAAGAGTTGCTCGGCGCTGATTTAATGGAACACCGGATTCGTCACTCACAAATCGGTTTATCGCGTGCCATTTCCGCACTAGCACCGATTAAGGTGAACCTCAAAGAAGTCGCTGGAATTCAGCCAATCGGTCTGAATCCAGGTCATGAGAAGAGCATTGAACAGCTACGCGCTGCCGAAGATAAATTATTAGAATGGCAGCGATATCTTGAACAAATGGGACCACATTTACCCAAGCATGTGCAAGAGCAATTCGCCACAGAGGAGCACAATATACTCAAAAAGATTCATACATCGCCCGACCCTAAGGCGCAAACGGTTGGCAATACTTTTAGTCACAAATTAAAAACGGTATATATGAAGAAAGACCCGGATCGCAATGTGCATACTCAAATTACTCCCGGGTTTTACAATCCGAACATTAAAGAATTACCAGTGATTTCAACGAAACAAATTGACAAGGATATCAATTTTGCATGGATCGATTGA

amino acid:

MTSKVTNVSASQATALKGIIRNSSYVIPGLYDLSVEDTNWVLTSSFIIFTMQTGFGMLESGCVSIKNEVNIMMKNVIDIVLGGFTYWLFGYGMSYGRGPLSNPFIAVGDFLLDPPVGDPLMGQIFAAFLFQLSFATTATTIVSGAMAERCNFKAYCIFSLLNTAVYCIPAGWVWGEHGFLNNLGAVDIAGSGPVHLIGGAAAFASAAMLGPRLGRYADGYDPLPLGNPVNACMGLFVLWWGWLAFNSGSTYGVSGAKWQYAARAAVMTMMGSFGGGIISCAYSLWRHDGRMDIIDLINGVLGSLVSITAGCFLYRAWEALVIGMLGALLCVVAMPLFDRLGVDDPVGASSVHGVCGIWGVIAVGLFADNPVPLETTKGRSGLFKGGGWYLVGIQTLSALCLACWGICSTFLLLYIINKFIPLRMDPNEELLGADLMEHRIRHSQIGLSRAISALAPIKVNLKEVAGIQPIGLNPGHEKSIEQLRAAEDKLLEWQRYLEQMGPHLPKHVQEQFATEEHNILKKIHTSPDPKAQTVGNTFSHKLKTVYMKKDPDRNVHTQITPGFYNPNIKELPVISTKQIDKDINFAWID-

**BcorAmt**

>amt trans

ATGACTTCAAAAGTGACAAATGTCTCAGCATCCCAAGCTACTGCGTTAAAGGGCATTATACGCAACAGCAGTTATGTCATACCAGGATTGTACGACCTTAGCGTGGAGGATACCAATTGGGTGTTGACGTCGTCGTTCATCATTTTCACAATGCAAACTGGGTTTGGCATGCTTGAGTCGGGTTGTGTATCGATTAAAAATGAGGTCAACATCATGATGAAGAACGTCATTGATATTGTACTTGGCGGCTTTACCTACTGGCTCTTCGGTTATGGCATGAGTTACGGCCGTGGACCACTTTCGAATCCATTCATAGCAGTTGGAGATTTTCTACTGGATCCACCTGTGGGTGATCCCTTAATGGGGCAAATTTTTGCTGCATTTTTATTTCAGTTGTCCTTTGCTACGACAGCAACAACGATTGTAAGTGGCGCCATGGCAGAAAGATGCAATTTCAAAGCGTACTGTATATTTTCGTTATTAAATACTGCTGTCTATTGTATACCCGCCGGTTGGGTGTGGGGTGAACATGGCTTCCTCAATAATCTTGGAGCTGTTGATATAGCGGGAAGTGGACCCGTTCATTTAATTGGAGGTGCAGCTGCATTTGCATCTGCTGCCATGTTGGGTCCACGTTTGGGTCGCTACGCAGACGGCTATGATCCTCTACCACTTGGTAATCCTGTGAACGCATGCATGGGTCTCTTTGTGCTCTGGTGGGGATGGCTAGCTTTTAATTCCGGCAGTACATATGGTGTAAGTGGTGCCAAATGGCAATATGCTGCACGTGCTGCTGTCATGACTATGATGGGTTCATTTGGTGGTGGTATCATAAGTTGCGCGTACTCGCTTTGGCGTCATGATGGGCGTATGGACATTATCGATCTTATTAACGGGGTACTCGGGTCACTGGTCTCAATTACGGCCGGGTGTTTTCTTTATCGTGCTTGGGAGGCACTAGTCATTGGGATGTTGGGTGCCTTACTTTGTGTTGTCGCAATGCCGTTGTTCGACCGGTTAGGAGTCGACGACCCAGTAGGTGCAAGCTCTGTTCACGGAGTTTGTGGCATTTGGGGTGTTATCGCGGTTGGACTTTTCGCTGATAATCCTATACCGTTAGAGACAACAAAGGGACGATCAGGACTATTTAAAGGTGGCGGTTGGTACTTGGTTGGCATACAGACTTTGTCGGCACTATGCCTTGCCTGCTGGGGCATATGTTCAACGTTTCTACTACTTTATATTATCAACAAATTTATACCATTACGCATGGATCCAAATGAAGAGTTGCTCGGCGCTGATTTAATGGAACACCGAATTCGTCACTCACAAATCGGTTTGTCCCGTGCCATTTCCGCACTGGCACCGATTAAGGTGAACCTCAAAGAAGTCGCCGGAATTCAGCCAATTGGTCTTAATCCAGGTCATGAGAAGAGCATTGAGCAGCTACGCGCCGCTGAAGATAAATTATTAGAGTGGCAGCGATATCTTGAACAAATGGGACCACATTTACCCAAGCATGTGCAAGAGCAATTCGCCACAGAGGAGCACAATATACTCAAAAAGATTCATACATCGCCCGATCCTAAGGCGCAAACGGTTGGCAATACTTTTAGTCACAAATTAAAAACGGTACATATCAAGAAAGACCCGGATCGTAATGTGCATACTCAAATTACTCCCGGGTTTTACAATCCGAACCTTAATGAATTACCAGTGATTTCAACGAAACAAATTGACAAGGATATCAATTTTGCATGGATCGATTGA

amino acid:

MTSKVTNVSASQATALKGIIRNSSYVIPGLYDLSVEDTNWVLTSSFIIFTMQTGFGMLESGCVSIKNEVNIMMKNVIDIVLGGFTYWLFGYGMSYGRGPLSNPFIAVGDFLLDPPVGDPLMGQIFAAFLFQLSFATTATTIVSGAMAERCNFKAYCIFSLLNTAVYCIPAGWVWGEHGFLNNLGAVDIAGSGPVHLIGGAAAFASAAMLGPRLGRYADGYDPLPLGNPVNACMGLFVLWWGWLAFNSGSTYGVSGAKWQYAARAAVMTMMGSFGGGIISCAYSLWRHDGRMDIIDLINGVLGSLVSITAGCFLYRAWEALVIGMLGALLCVVAMPLFDRLGVDDPVGASSVHGVCGIWGVIAVGLFADNPIPLETTKGRSGLFKGGGWYLVGIQTLSALCLACWGICSTFLLLYIINKFIPLRMDPNEELLGADLMEHRIRHSQIGLSRAISALAPIKVNLKEVAGIQPIGLNPGHEKSIEQLRAAEDKLLEWQRYLEQMGPHLPKHVQEQFATEEHNILKKIHTSPDPKAQTVGNTFSHKLKTVHIKKDPDRNVHTQITPGFYNPNLNELPVISTKQIDKDINFAWID-

**BcucAmt**

>amt trans

ATGACTTCAAAAGTGACAAATGTCTCAGCATCACAAGCCACAGCGTTGAAGGGCATTATACGCAATAGTAGTTATGTCATACCAGGATTATACGATCTTAGCGTGGAGGACACCAATTGGGTATTGACGTCATCCTTCATAATTTTCACAATGCAAACAGGATTTGGCATGCTCGAATCGGGTTGTGTATCAATTAAAAATGAGGTCAACATTATGATGAAGAACGTCATTGATATTGTACTTGGCGGCTTTACCTACTGGCTCTTCGGGTATGGCATGAGTTACGGGCGTGGACCACTTTCGAATCCATTCATAGCGGTTGGAGATTTTCTACTTGATCCACCTGTCGGCGATCCCTTGATGGGACAAATTTTTGCTGCCTTTTTATTTCAGCTGTCCTTCGCTACAACAGCAACCACGATTGTAAGTGGCGCCATGGCAGAAAGATGCAATTTCAAAGCGTACTGTTTATTTTCGTTACTAAATACTGCTGTCTACTGTATACCCGCCGGTTGGGTGTGGGGTGAGCATGGTTTCCTCAATAATCTTGGAGCTGTTGATATAGCCGGAAGTGGGCCCGTACATTTAATTGGAGGTGCCGCCGCATTTGCATCTGCAGCTATGTTAGGTCCGCGGTTGGGTCGCTACTCGGACGGCTATGATCCTCTACCACTTGGTAATCCAGTGAACGCATGTATGGGTCTGTTTGTGCTCTGGTGGGGTTGGCTTGCTTTCAATTCCGGCAGTACCTATGGTGTAAGTGGAGCCAAATGGCAATATGCGGCACGTGCCGCTGTCATGACTATGATGGGTTCATTTGGAGGTGGGATCATAAGTTGCGCGTACTCGCTTTGGCGCCATGATGGACGTATGGACATTATCGACCTTATTAACGGTGTGCTCGGGTCACTGGTCTCAATTACGGCTGGATGTTTCCTATATCGTGCTTGGGAGGCACTAGTCATTGGTATGTTGGGTGCACTGCTCTGTGTTGTCGCAATGCCTTTGTTCGACCGGTTAGGGGTGGATGACCCAGTAGGTGCCAGCTCGGTTCATGGTGTTTGTGGAATTTGGGGCGTTCTCGCGGTCGGATTTTTCGCTGATAATCCTGTACCGCTTGAGACAACCAAGGGACGTTCAGGCCTATTTAAAGGTGGTGGTTGGTACTTGATCGGCATACAGAGTTTATCGGCATTATGTCTTGCTTGCTGGGGCATATGTTCAACATTTCTACTACTTTACATTATCAATAAATTTATACCTTTGCGCATGGACCCCAATGAAGAGTTGCTCGGCGCTGATCTTATGGAACATCGGATTCGTCACTCACAAATCGGGTTGTCGCGTGCCATTTCCGCACTTGCACCGATTAAGGTGAATCTCAAGGAAGTCGCCGGAATTCAACCGATTGGCCTAAATCCAGGTCACGAGAAAAGTATTGAACAACTACGCGCTGCCGAAGATAAATTACTAGAATGGCAGCAATATCTAGAACAAATGGGTCCACAATTACCCAAACATGTGCAAGAGCAATTTGCCACAGAGGAGCACAATATACTCAAGAAGATTCATAATTCGCCTGATCCCAAAGCGCAAACGATTGGCAATGTTTTTAGTAACAAATTAAAATCAGTACATATCAAGAAAGATCCAGACCGCAATGTGCATACCCAAATTACTCCCGGCTTTTATAAACCGAACAATAAAGAATTGCCAGTAATTTCGTCAAGACAAATTGACAAGGATACCAATTTCGCATGGATCGATTGA

amino acid:

MTSKVTNVSASQATALKGIIRNSSYVIPGLYDLSVEDTNWVLTSSFIIFTMQTGFGMLESGCVSIKNEVNIMMKNVIDIVLGGFTYWLFGYGMSYGRGPLSNPFIAVGDFLLDPPVGDPLMGQIFAAFLFQLSFATTATTIVSGAMAERCNFKAYCLFSLLNTAVYCIPAGWVWGEHGFLNNLGAVDIAGSGPVHLIGGAAAFASAAMLGPRLGRYSDGYDPLPLGNPVNACMGLFVLWWGWLAFNSGSTYGVSGAKWQYAARAAVMTMMGSFGGGIISCAYSLWRHDGRMDIIDLINGVLGSLVSITAGCFLYRAWEALVIGMLGALLCVVAMPLFDRLGVDDPVGASSVHGVCGIWGVLAVGFFADNPVPLETTKGRSGLFKGGGWYLIGIQSLSALCLACWGICSTFLLLYIINKFIPLRMDPNEELLGADLMEHRIRHSQIGLSRAISALAPIKVNLKEVAGIQPIGLNPGHEKSIEQLRAAEDKLLEWQQYLEQMGPQLPKHVQEQFATEEHNILKKIHNSPDPKAQTIGNVFSNKLKSVHIKKDPDRNVHTQITPGFYKPNNKELPVISSRQIDKDTNFAWID-

**BtauAmt**

>amt trans

ATGACTTCAAAAGTGACAAATGTCTCAGCATCACAAGCCACAGCGTTGAAGGGTATTATACGCAATAGTAGTTATGTCATACCAGGATTATACGATCTTAGCGTGGAGGACACCAATTGGGTATTGACGTCATCCTTTATAATTTTCACAATGCAAACAGGATTTGGCATGCTCGAATCGGGTTGTGTATCAATTAAAAATGAGGTCAACATTATGATGAAGAACGTCATTGATATTGTACTTGGCGGCTTTACCTACTGGCTTTTCGGGTATGGCATGAGTTACGGGCGTGGACCACTTTCGAATCCATTCATAGCGGTTGGAGATTTTCTACTTGATCCACCTGTCGGCGACCCCTTGATGGGACAAATTTTCGCTGCCTTTTTATTTCAGCTGTCCTTCGCTACAACAGCAACCACGATTGTAAGTGGCGCCATGGCAGAAAGATGCAATTTCAAAGCGTACTGTTTATTTTCGTTACTAAATACTGCTGTCTACTGTATACCCGCCGGTTGGGTGTGGGGTGAGCATGGTTTCCTCAATAATCTTGGAGCTGTTGATATAGCCGGAAGTGGGCCCGTACATTTAATTGGAGGTGCCGCCGCATTTGCATCTGCAGCTATGTTAGGTCCGCGGTTGGGTCGCTACTCGGACGGCTATGATCCTCTACCACTTGGTAATCCAGTGAACGCATGTATGGGTCTGTTTGTGCTCTGGTGGGGTTGGCTCGCTTTCAATTCCGGCAGTACCTATGGTGTAAGTGGAGCCAAATGGCAATATGCGGCACGTGCCGCTGTCATGACTATGATGGGTTCATTTGGAGGTGGGATCATAAGTTGCGCGTACTCGCTTTGGCGTCATGATGGACGTATGGACATTATCGACCTTATTAACGGTGTGCTCGGTTCACTGGTCTCAATTACGGCTGGATGTTTCCTATATCGTGCTTGGGAGGCACTAGTCATTGGTATGTTGGGTGCACTGCTCTGTGTTGTCGCAATGCCTTTGTTCGACCGGTTAGGGGTGGATGACCCAGTAGGTGCCAGCTCGGTTCATGGTGTTTGTGGAATTTGGGGCGTTCTCGCGGTCGGATTTTTCGCTGATAATCCTGTACCGCTAGAGACAACCAAGGGACGTTCAGGCCTATTTAAAGGTGGTGGTTGGTACTTGATCGGCATACAGAGTTTATCGGCGTTATGTCTTGCTTGCTGGGGCATATGCTCAACATTTCTACTACTTTACATTATCAATAAATTTATACCTTTGCGCATGGATCCCAATGAAGAGTTGCTCGGCGCTGATCTAATGGAACATCGGATTCGTCACTCACAAATCGGGTTGTCGCGTGCCATTTCCGCACTTGCACCGATTAAGGTGAATCTCAAGGAAGTCGCCGGAATTCAACCGATTGGCCTAAATCCAGGTCACGAGAAAAGTATTGAACAACTACGCGCTGCCGAAGATAAATTACTAGAATGGCAGCAATATCTAGAACAAATGGGTCCACAATTACCCAAACATGTGCAAGAGCAATTTGCCACAGAGGAGCACAATATACTCAAGAAGATTCATAATTCGCCTGATCCCAAAGCGCAAACGATTGGCAATGTTTTTAGTAACAAATTAAAATCAGTACACATCAAGAGAGACCCGGACCGTAACGTACATACCCAAATTACTCCCGGCTTTTATAAACCGAACAATAAAGAATTGCCAGTAATTTCGTCGAGACAAATTGACAAGGATACCAATTTCGCATGGATCGATTGA

amino acid:

MTSKVTNVSASQATALKGIIRNSSYVIPGLYDLSVEDTNWVLTSSFIIFTMQTGFGMLESGCVSIKNEVNIMMKNVIDIVLGGFTYWLFGYGMSYGRGPLSNPFIAVGDFLLDPPVGDPLMGQIFAAFLFQLSFATTATTIVSGAMAERCNFKAYCLFSLLNTAVYCIPAGWVWGEHGFLNNLGAVDIAGSGPVHLIGGAAAFASAAMLGPRLGRYSDGYDPLPLGNPVNACMGLFVLWWGWLAFNSGSTYGVSGAKWQYAARAAVMTMMGSFGGGIISCAYSLWRHDGRMDIIDLINGVLGSLVSITAGCFLYRAWEALVIGMLGALLCVVAMPLFDRLGVDDPVGASSVHGVCGIWGVLAVGFFADNPVPLETTKGRSGLFKGGGWYLIGIQSLSALCLACWGICSTFLLLYIINKFIPLRMDPNEELLGADLMEHRIRHSQIGLSRAISALAPIKVNLKEVAGIQPIGLNPGHEKSIEQLRAAEDKLLEWQQYLEQMGPQLPKHVQEQFATEEHNILKKIHNSPDPKAQTIGNVFSNKLKSVHIKRDPDRNVHTQITPGFYKPNNKELPVISSRQIDKDTNFAWID

## References

[1] Shelly T., Epsky N., Jang E.B., Reyes-Flores J., Vargas R. (2014). Trapping and the Detection, Control, and Regulation of Tephritid Fruit Flies.

[2] Christenson L.D., Foote R.H. (1960). Biology of fruit flies. Annu. Rev. Entomol..

[3] Ono H., Hee A.K.W., Jiang H.B. (2021). Recent advancements in studies on chemosensory mechanisms underlying detection of semiochemicals in Dacini fruit flies of economic importance (Diptera: Tephritidae). Insects.

[4] Smith P.T., Kambhampati S., Armstrong K.S. (2003). Phylogenetic relationships among *Bactrocera* species (Diptera: Tephritidae) inferred from mitochondrial DNA sequences. Mol. Phylogenet. Evol..

[5] Bateman M.A., Morton T.C. (1981). The importance of ammonia in proteinaceous attractants for fruit flies (Diptera: Tephritidae). Aust. J. Agric. Res..

[6] Mazor M., Peysakhis A., Reuven G. (2002). Release rate of ammonia-a key component in the attraction of female mediterranean fruit fly to protein-based food lures. IOBC-WPRS Bull..

[7] Epsky N.D., Heath R.R. (1998). Exploiting the interactions of chemical and visual cues in behavioral control measures for pest Tephritid fruit flies. Flo. Entomol..

[8] Robacker D., Flath R. (1995). Attractants from *Staphylococcus aureus* cultures for mexican fruit fly, *Anastrepha ludens*. J. Chem. Ecol..

[9] Robacker D.C., Heath R.R. (1996). Attraction of mexican fruit flies (Diptera: Tephritidae) to lures emitting host-fruit volatiles in a citrus orchard. Flo. Entomol..

[10] Hull C., Cribb B. (2001). Olfaction in the Queensland Fruit Fly, *Bactrocera tryoni*. I: Identification of olfactory receptor neuron types responding to environmental odors. J. Chem. Ecol..

[11] Phyoe A., Nwet T.T., Maung K.L. (2020). Effectiveness of protein bait on the attraction and reproduction of *Bactrocera dorsalis* and *Bactrocera correcta* (Diptera: Tephritidae). Univ. J. Sci. Eng. Res..

[12] Drew R., Courtice A.C., Teakle A. (1983). Bacteria as a natural source of food for adult fruit flies (Diptera: Tephritidae). Oecologia.

[13] Hagen K.S., Finney G.L. (1950). A food supplement for effectively increasing the fecundity of certain tephritid species. J. Econ. Entomol..

[14] Biasazin T.D., Chernet H.T., Herrera S.L., Bengtsson M., Karlsson M.F., Lemmen-Lechelt J.K., Dekker T. (2018). Detection of volatile constituents from food lures by Tephritid fruit flies. Insects.

[15] Piñero J.C., Mau R.F.L., Vargas R.I. (2011). A comparative assessment of the response of three fruit fly species (Diptera: Tephritidae) to a spinosad-based bait: Effect of ammonium acetate, female age, and protein hunger. Bull. Entomol. Res..

[16] Piñero J.C., Souder S.K., Smith T.R., Vargas R.I. (2017). Attraction of *Bactrocera cucurbitae* and *Bactrocera dorsalis* (Diptera: Tephritidae) to beer waste and other protein sources laced with ammonium acetate. Fla. Entomol..

[17] Bruce T.J.A., Wadhams L.J., Woodcock C.M. (2005). Insect host location: A volatile situation. Trends Plant Sci..

[18] Montagné N., de Fouchier A., Newcomb R.D., Jacquin-Joly E. (2015). Advances in the identification and characterization of olfactory receptors in insects. Prog. Mol. Biol. Transl. Sci..

[19] Menuz K., Larter N.K., Park J., Carlson J.R. (2014). An RNA-seq screen of the *Drosophila antenna* identifies a transporter necessary for ammonia detection. PLoS Genet..

[20] Pitts R.J., Derryberry S.L., Pulous F.E., Zwiebel L.J. (2014). Antennal-expressed ammonium transporters in the malaria vector mosquito *Anopheles gambiae*. PLoS ONE.

[21] Vulpe A., Kim H.S., Ballou S., Wu S.T., Grabe V., Nava Gonzales C., Liang T., Sachse S., Jeanne J.M., Su C.Y. (2021). An ammonium transporter is a non-canonical olfactory receptor for ammonia. Curr. Biol..

[22] Khademi S., Stroud R.M. (2006). The Amt/MEP/Rh family: Structure of AmtB and the mechanism of ammonia gas conduction. Physiology.

[23] Zheng L., Kostrewa D., Bernèche S., Winkler F.K., Li X.D. (2004). The mechanism of ammonia transport based on the crystal structure of AmtB of *Escherichia coli*. Proc. Natl. Acad. Sci. USA.

[24] Khademi S., O’Connell J., Remis J., Robles-Colmenares Y., Miercke L.J., Stroud R.M. (2004). Mechanism of ammonia transport by Amt/MEP/Rh: Structure of AmtB at 1.35 A. Science.

[25] Maniero R.A., Koltun A., Vitti M., Factor B.G., de Setta N., Câmara A.S., Lima J.E., Figueira A. (2023). Identification and functional characterization of the sugarcane (*Saccharum* spp.) AMT2-type ammonium transporter ScAMT3;3 revealed a presumed role in shoot ammonium remobilization. Front. Plant Sci..

[26] Delventhal R., Menuz K., Joseph R., Park J., Sun J.S., Carlson J.R. (2017). The taste response to ammonia in *Drosophila*. Sci. Rep..

[27] Grabherr M.G., Haas B.J., Yassour M., Levin J.Z., Thompson D.A., Amit I., Adiconis X., Fan L., Raychowdhury R., Zeng Q. (2011). Full-length transcriptome assembly from RNA-Seq data without a reference genome. Nat. Biotechnol..

[28] Emms D.M., Kelly S. (2019). OrthoFinder: Phylogenetic orthology inference for comparative genomics. Genome. Biol..

[29] Abramson J., Adler J., Dunger J., Evans R., Green T., Pritzel A., Ronneberger O., Willmore L., Ballard A.J., Bambrick J. (2024). Accurate structure prediction of biomolecular interactions with AlphaFold 3. Nature.

[30] DeLano W.L. (2002). Pymol: An open-source molecular graphics tool. CCP4 Newsl. Protein Crystallogr..

[31] Katoh K., Standley D.M. (2013). MAFFT multiple sequence alignment software version 7: Improvements in performance and usability. Mol. Biol. Evol..

[32] Minh B.Q., Schmidt H.A., Chernomor O., Schrempf D., Woodhams M.D., von Haeseler A., Lanfear R. (2020). IQ-TREE 2: New models and efficient methods for phylogenetic inference in the genomic era. Mol. Biol. Evol..

[33] Audic S., Claverie J.M. (1997). The significance of digital gene expression profiles. Genome Res..

[34] Mortazavi A., Williams B.A., McCue K., Schaeffer L., Wold B. (2008). Mapping and quantifying mammalian transcriptomes by RNA-Seq. Nat. Methods.

[35] Andersson M.N., Videvall E., Walden K.K.O., Harris M.O., Robertson H.M., Lofstedt C. (2014). Sex- and tissue-specific profiles of chemosensory gene expression in a herbivorous gall-inducing fly (Diptera: Cecidomyiidae). BMC Genom..

[36] Leitch O., Papanicolaou A., Lennard C., Kirkbride K.P., Anderson A. (2015). Chemosensory genes identified in the antennal transcriptome of the blowfly *Calliphora stygia*. BMC Genom..

[37] Love M.I., Huber W., Anders S. (2014). Moderated estimation of fold change and dispersion for RNA-seq data with DESeq2. Genome Biol..

[38] Wang L., Feng Z., Wang X., Wang X., Zhang X. (2010). DEGseq: An R package for identifying differentially expressed genes from RNA-seq data. Bioinformatics.

[39] Livak K.J., Schmittgen T.D. (2001). Analysis of relative gene expression data using real-time quantitative PCR and the 2(-Delta Delta C(T)) method. Methods.

[40] Gruswitz F., Chaudhary S., Ho J.D., Schlessinger A., Pezeshki B., Ho C.M., Sali A., Westhoff C.M., Stroud R.M. (2010). Function of human Rh based on structure of RhCG at 2.1 A. Proc. Natl. Acad. Sci. USA.

[41] Schlessinger A., Yee S.W., Sali A., Giacomini K.M. (2013). SLC classification: An update. Clin. Pharmacol. Ther..

[42] Zhang X.X., Liu Y., Guo M.B., Sun D.D., Zhang M.J., Chu X., Berg B.G., Wang G.R. (2024). A female-specific odorant receptor mediates oviposition deterrence in the moth *Helicoverpa armigera*. Curr. Biol..

[43] Zhang R.B., Wang B., Grossi G., Falabella P., Liu Y., Yan S.C., Lu J., Xi J.H., Wang G.R. (2017). Molecular basis of alarm pheromone detection in aphids. Curr. Biol..

[44] Pitts R.J., Fox A.N., Zwiebel L.J. (2004). A highly conserved candidate chemoreceptor expressed in both olfactory and gustatory tissues in the malaria vector *Anopheles gambiae*. Proc. Natl. Acad. Sci. USA.

[45] Brigaud I., Montagné N., Monsempes C., François M.C., Jacquin-Joly E. (2009). Identification of an atypical insect olfactory receptor subtype highly conserved within noctuids. FEBS J..

[46] Wang Q., Wang Q., Zhou Y.L., Shan S., Cui H.H., Xiao Y., Dong K., Khashaveh A., Sun L., Zhang Y.J. (2018). Characterization and comparative analysis of olfactory receptor co-receptor Orco orthologs among five mirid bug species. Front. Physiol..

[47] Hallem E.A., Ho M.G., Carlson J.R. (2004). The molecular basis of odor coding in the *Drosophila antenna*. Cell.

[48] Chang H.T., Cassau S., Krieger J., Guo X.J., Knaden M., Kang L., Hansson B.S. (2023). A chemical defense deters cannibalism in migratory locusts. Science.

